# La Grippe

**Published:** 1890-03

**Authors:** 


### LA GRIPPE.

Dr. Edward T. Balch, of South Bend, in the new State of Washington, writes to us thus:

"La grippe has been in full force here. In fifty cases not a death, or, indeed, any sequalæ. It yields readily to the potentized remedy, the most frequently indicated being, in order, Naphthaline, Gelsem., Acon., Ars., and, in three cases, Lycopod."

